# Recognizing the ethical complexity of food policies and the role of the food industry

**DOI:** 10.1093/heapro/daae168

**Published:** 2024-11-28

**Authors:** Safura Abdool Karim, Miriam Alvarado, Tess Johnson, Anne Barnhill

**Affiliations:** Berman Institute of Bioethics, Johns Hopkins University, 1809 Ashland Avenue, Baltimore, MD 21205, USA; Center for the Aids Programme of Research in South Africa, Doris Duke Medical Research Institute (DDMRI), University of KwaZulu Natal, 719 Umbilo Road, Durban, 4001, South Africa; MRC Epidemiology Unit, University of Cambridge, Level 3 Institute of Metabolic Science, University of Cambridge School of Clinical Medicine, Cambridge, CB2 0SL, UK; Nuffield Department of Population Health, Ethox Centre, University of Oxford, Rosemary Rue Building, Old Road Campus, Headington, Oxford, OX37LF, UK; Pandemic Sciences Institute, University of Oxford, Old Road Campus Research Building, Old Road Campus, Roosevelt Drive, Oxford, OX3 7BN, UK; Berman Institute of Bioethics, Johns Hopkins University, 1809 Ashland Avenue, Baltimore, MD 21205, USA; Department of Health Policy and Management, Bloomberg School of Public Health, Johns Hopkins University, 615 N Wolfe St, Baltimore, MD 21205, USA

**Keywords:** ethical analysis, food policy, SSB taxation, industry response, bioethics

## Abstract

Restrictive food policies are often contentious and controversial. Supporters of these policies view them as imperative for achieving public health aims while some opponents view them as overly paternalistic, infringing on consumer choice and potentially inequitable. As a consequence, their ethical status and permissibility are both contested and of importance in decision-making for policy. Traditional ethical analysis of these interventions has examined the ethical implications of the policies according to a direct, linear view of the relationships between government and consumer and the impact of government policy on the consumer. However, this approach to ethical analysis fails to take into account the role of the food industry as the subjects of the policies and intermediaries between government and consumers in the implementation and effectiveness of the policies. The actions of the food industry in response to a policy substantially determine how the policy translates to changes in the food supply and thus, the effect of the policy on consumers. This has significant implications for the ethical status of the policy. As a result, this article calls for complicating the common ethical approach to restrictive food policies by adopting a framing that recognizes the role of the food industry in the implementation of these policies. We then discuss three implications this framing has for ethical analysis: first that ethical analysis must be more nuanced and recognize the potentially complex outcomes of a policy, second that it must be dynamic and ongoing and third that underlying assumptions about policies’ effects on choice, effectiveness and equity need to be reconsidered.

**Contribution to Health Promotion**

- Current approaches to ethical analysis do not adequately recognize the complexity of how food policies are implemented.
- There is a need to better recognize the role the food industry plays in how policies are implemented and the effect they have on food supply and consumer demand.
- By recognizing that restrictive food policy may yield a wide range of potential outcomes, ethical analysis can become more nuanced and empirically informed.
- Adopting a framing which includes industry as an active influence on the implementation of policy also enables us to rethink assumptions about consumer choice, effectiveness and equity in food policy.

## INTRODUCTION

Diet-related diseases are an increasingly concerning issue in global health. Rates of obesity and associated adverse health outcomes are already high in many high-income countries, and many low- and middle-income countries are experiencing growing epidemics of non-communicable diseases (NCDs) (Habib and Saha, 2010; Benziger *et al.*, 2016). These diseases and other poor health outcomes are linked to the consumption of unhealthy products including alcohol, tobacco and unhealthy foods (such as ultra-processed foods or foods high in saturated fat, salt and sugar) (Benziger *et al.*, 2016). When it comes to food, the food system and changes in nutrition are recognized to be significant contributors to these diseases (Popkin and Ng, 2022). These changes and the increased consumption of unhealthy foods are often driven by commercial actors who profit from the consumption of these products—leading to some terming the rise of diet-related disease an ‘industrial epidemic’ (Collin and Hill, 2015). The proliferation of unhealthy food is also recognized as part of the commercial determinants of health (Gilmore *et al*., 2023).

How consumers make choices about food is complex and influenced by a range of factors (Chen and Antonelli, 2020). Some of these are individualized and internal to a consumer, such as a consumer’s taste and preferences, and physiological needs (Chen and Antonelli, 2020). Others are socio-cultural, driven by a person’s political, cultural and economic environment. Others still are external, linked to the social and physical food environment (Chen and Antonelli, 2020). Addressing the internal influences on food choice was previously the preferred mechanism to address diet-related disease, but over the past two decades, there has been a call to shift to structural interventions targeting the food system, specifically unhealthy foods, as a mechanism to prevent diet-related disease (Yang *et al.*, 2018). As a consequence, many countries have begun adopting interventions aimed at reducing the consumption of unhealthy foods, with the underlying goal of preventing NCDs (Lyn *et al.*, 2019).

While there is a broad spectrum of interventions which can be used to respond to the NCD epidemic, restrictive food policies that aim to limit the availability, affordability and acceptability of unhealthy foods are increasingly being adopted (Fox and Horowitz, 2013; Zhang *et al.*, 2014). This includes measures such as taxation on sugar-sweetened beverages (SSBs) and unhealthy foods, front-of-package warning labels and restrictions on the marketing and sales of unhealthy foods to particular populations, such as limiting child-directed marketing or sales of SSBs in schools (Swinburn, 2008; Fox and Horowitz, 2013). These interventions, which are the focus of this article, target and often seek to reduce consumption of key ingredients such as sodium, sugar and saturated or trans-fats as a means to improve public health.

Notwithstanding the increased adoption of these measures, many of them remain ethically and politically contentious, even among their proponents (Fry, 2012; Upshur, 2013; Barnhill and Civita, 2020). Opponents of some of these measures, such as taxes or restrictions on selling certain products, typically assume that they will negatively affect consumer choice—either by eliminating the sale of a product entirely or by causing a price increase that will prevent some consumers from having their preferred level of consumption. Limiting consumer choice in these ways to improve consumers’ heath is critiqued as an unjustifiably paternalistic limitation on liberty and an infringement on consumers’ autonomy by some who believe that consumers can and should make their own choices about their food consumption and health without state interference. Policies such as taxes are also ethically critiqued as inequitable, because they have a relatively larger financial impact on lower-income people who may also often have reduced access to healthy foods (Barnhill and King, 2013a, 2013b; Barnhill, 2015). However, other public health advocates and ethicists have argued that the measures are justified. For some, this is due to their benefiting not only individual health but also reducing the burden on healthcare systems (Bombak, 2014). For others, these ‘systemic interventions’ which target the food environment rather than individuals directly, may be less paternalistic and coercive and more effective than alternative measures (Paetkau, 2024), although the view that systemic interventions are necessarily less coercive has been contested (Johnson, 2024). For some, this increased effectiveness may also outweigh concerns about the measures’ inequitable effects. In particular, substantial literature has outlined the ethical implications of SSB taxation and front-of-package labelling, finding the measures to be acceptable, notwithstanding the resulting infringements of autonomy and other potential harms that result from the interventions (Greer and Ryckeley, 2011; Fry, 2012; Buchanan, 2013; Bonotti, 2014; Ten Have, 2014; Barnhill, 2015; Goranova-Spasova *et al.*, 2018; Grummon *et al.*, 2020; Thompson, 2023).

**Fig. 1:**
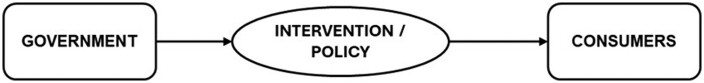
Conceptualization of food policy interventions in typical ethical analysis.

Among both proponents and opponents of these policies, analysis is confined to the relationship between governments or state actors and their citizens (see Figure 1). In doing so, ethical analyses to date have framed restrictive food policies as being measures that emanate from the government and produce effects or impacts felt directly by consumers (Bartlett, 2018; Steele *et al.*, 2020). In this regard, common ethical analysis of food policies is premised on the government being the authority figure that implements public health measures for the benefit of the public and citizens. Given that most of the existing ethical analysis adopts this frame, we do not wish to investigate this relationship but rather use it as a point of departure.

This framing of intervention as between government and citizen has a central shortcoming in that it misses a key component of how these interventions are operationalized and implemented, and where some important ethical implications arise—namely, how the food industry (including retailers, manufacturers and producers) responds to the intervention (thereby further affecting consumers). Specifically, this conceptualization fails to recognize the complexity of these interventions and thus the range of ethical implications an intervention may have. For example, Grummons *et al.* outline, in detail, the ethical implications of products carrying warning labels on the assumption that the consequence of adopting a FOPL policy would be that products carry the labels which consumers then react to. While they recognize that one of the purposes of the policy is to spur reformulation, the ethical implications of reformulation are not considered or discussed beyond noting the possibility of this happening (Grummon *et al*., 2020). However, reformulation and product changes are commonplace responses to these policies and can have even more significant effects on health than the direct policy-to-consumer route of intervention. They may also result in changes to dietary intake (or the desired reductions in consumption) without necessarily requiring independent changes in consumer behaviour (Hashem *et al.*, 2017). Additionally, this oversight in turn leads to these analyses overlooking the food industry as a potentially influential and powerful actor in both shaping food policies and their implementation (Nestle, 2002).

This article seeks to reframe these food policies as involving a triad of actors—governments, consumers and the food industry—and to discuss the implications this reframing has on ethical analysis of, in particular, restrictive food policies.

## HOW DO RESTRICTIVE FOOD POLICIES OPERATE AS PUBLIC HEALTH INTERVENTIONS?

As described above, food environments are complex, consisting of a number of components that operate at different levels to influence dietary behaviours and choices (Constantinides *et al*., 2021). While internal individual, cultural and other personal factors do shape diet, these are not usually the focus of policies targeting unhealthy food (Constantinides *et al*., 2021). Rather the policies focus on the external aspects of the food environment such as food supplies, availability and affordability (Osei-Kwasi *et al*., 2021).

These interventions operate by identifying unhealthy foods or (more typically) harmful levels of certain ingredients (such as sugar or sodium or multiple key ingredients) and implementing policies targeting the unhealthy product or products with these ingredients across the food system, with the aim of producing population-level public health benefits. The policy adopted, such as a tax, warning labelling schemes or marketing restrictions can sometimes restrict the presence of the ingredient in products either completely (as is the case for bans on trans-fats), or by setting a maximum threshold for the key ingredient’s presence (as is the case with tiered SSB taxes). This approach is not unique to NCD-related dietary risk factors—a similar process is also used to regulate other harmful ingredients as a part of food safety laws, and is sometimes used for other unhealthy commodities such as alcohol.

What is important is that the consequences of the policy are not automatic. The producer who manufactures products that are targeted by the policy has one or a number of choices: remove or reduce the amount of the ingredient to below the threshold, introduce new or changed products which may be cheaper or more compliant with the policy, or be subject to the consequences of the policy. In exercising this choice, the food industry then holds a fair degree of power in shaping the public health consequence of the policy (Lyn *et al.*, 2019). Therefore, the effect of the policy may differ by product or manufacturer and the consequences may play out differently. First, they may be felt directly by a consumer (through, for example, a price change); second, they may enhance consumer choice by introducing a greater range of choices (through new product offerings); third, they may result in individual choices being impacted through higher prices or by reducing the available options by removing products from stores when non-compliant (this was seen in response to sodium restrictions when industry could not sufficiently reduce the amount of sodium while retaining the palatability of the product).

In this way, food industry actors play a substantial role in how restrictive policies translate into changes (or not) in the food system (Binks, 2016) and public health (Ng *et al.*, 2022), because the interventions are actually targeting the food producers and manufacturers who then shape the food environment through their conduct (Binks, 2016). In other words, industry has the power to determine whether and how these policies accomplish their intended aims, and in doing so, has the power to affect the relationship between citizen and state and the state’s ability to shape citizens’ health. It ought to be acknowledged that this translation is not agnostic. As research has shown that the food industry may act as a barrier to both the implementation of food policies and achieving the public health aims of such policies (Lyn *et al.*, 2019; Ng *et al.*, 2022). Consequently, we note that the food industry is not a neutral recipient of these policies and, in translating policies, may act in particular ways that protect their interests or undermine public health though we do not assume this will always be the case.

The manner in which industry shapes the implementation and results of a policy can be observed in responses to policies on the taxation of sugary beverages. Almost all countries that have implemented a sugar tax have done so through the mechanism of an excise tax (Backholer *et al.*, 2018; Fiscal Policies For Health, 2022). Some, like the UK and South Africa, employ tiered designs—applying higher taxes as the sugar content increases while others like Barbados Mexico apply a flat or uniform tax on all products containing added sugar (Backholer *et al*., 2018; Barbados Raises Sugary Drinks Tax to Fight Disease, 2022). These excise taxes are levied on manufacturers at the point of production. Manufacturers can then decide whether to pass this cost on to distributors or retailers, who can also decide whether to pass on the cost (or portions thereof) to consumers or absorb it, keeping the price of their product the same and reducing their profits (Veerman, 2017). Some elect to do the latter, specifically to avoid price-related reductions in purchases of their product. Alternatively, producers can reformulate their products or change their product lines to introduce new products which are not subject to tax (Veerman, 2017). With tiered taxes, companies can even choose to avoid the tax altogether by reducing the amount of sugar in their products—as was observed in a number of products in the UK and South Africa (Stacey *et al*., 2019; Scarborough *et al*., 2020). If sugary drink producers bring the sugar content of their products under the threshold then there is no impact on the end price to consumers as a result of the tax but there is a change in the product’s nutritional composition—a distinction which is ethically significant.

All of these responses, often in combination, have seen some uptake by beverage producers in countries that have a tax. Yet, despite this array of potential responses, ethical analyses of SSB taxation have been focused on the outcome in which taxes directly impact consumers by increasing the prices of SSBs. In failing to acknowledge the range of possible responses by industry and consequent outcomes for consumers, ethical analyses are overly simplistic, because these outcomes may be significantly ethically different, as discussed below.

We have discussed in substantial detail the example of an SSB taxation but a similar process holds for other kinds of restrictive food policies. For example, front-of-package warning labels or other interpretive labelling schemes are another policy which has seen increasing uptake. In the case of warning labels, the government identifies harmful ingredients and determines thresholds for these ingredients in foodstuffs (Kanter *et al.*, 2018). Products which exceed these thresholds must carry a warning label—that is the extent of the government’s intervention—yet there are a variety of changes these schemes may cause in the food supply (Kanter *et al.*, 2018). Food producers with products subject to this regulation and may elect to sell their products with the relevant warning labels, to reformulate their products so that they are not required to carry a warning label or to change their product offerings to either remove products that carry labels or increase the range of products that are not required to carry a warning label (Flexner *et al*., 2023). Again, the effect these elections have on consumers is variable: it may result in a greater product range being available, or consumers being subjected to interpretive warning labels on unhealthy products, or in their finding fewer products offered or in the composition of particular products changing (Muzzioli *et al*., 2022; Braesco and Drewnowski, 2023). There are different and distinct ethical implications to each of these elections. A comprehensive ethical analysis would consider this suite of potential outcomes and their permissibility according to where their harms and benefits are targeted, how likely they are to reduce consumption of harmful ingredients, and whether they have other unintended consequences (e.g. greater general nutritional awareness, or higher spending on alternative foods). Food retailers may also be influenced by these policies and may change the range of products they carry or where these products are located within their stores to prioritize the visibility of products without labels. There are, then, a few degrees of separation between the policy governments implement and the end impact it has on consumers. Moreover, the impact the policy has on consumers is primarily determined by the food industry, not the government.

To be clear, the role of industry actors in shaping food environments and contributing to unhealthy diets has been noted by ethicists. For example, ten Have *et al*. acknowledge that industry actors bear a portion of responsibility in the ‘complex web of causal factors’ that have contributed to the obesity epidemic (Ten Have *et al*., 2013). What needs to also be acknowledged and incorporated into the ethical analysis of specific policies, or so we argue, is that these policies may result in different market outcomes and public health outcomes depending upon the actions of the industry. For example, Ten Have *et al*. (2013) offer a framework for thinking through the ethics of overweight and obesity interventions, which consists of eight questions about their morally relevant features. The first question asks, ‘How does the programme affect physical health?’ and ten Have *et al*. identify several considerations to take into account when answering this question, including whether scientific evidence about the intervention is available and whether the intervention may encourage unhealthy weight loss methods. What is not included, but should be, is consideration of the active role of industry and how this affects the implementation of the policy.

## THE TRIAD OF ACTORS AND ETHICAL OBLIGATIONS

**Fig. 2:**
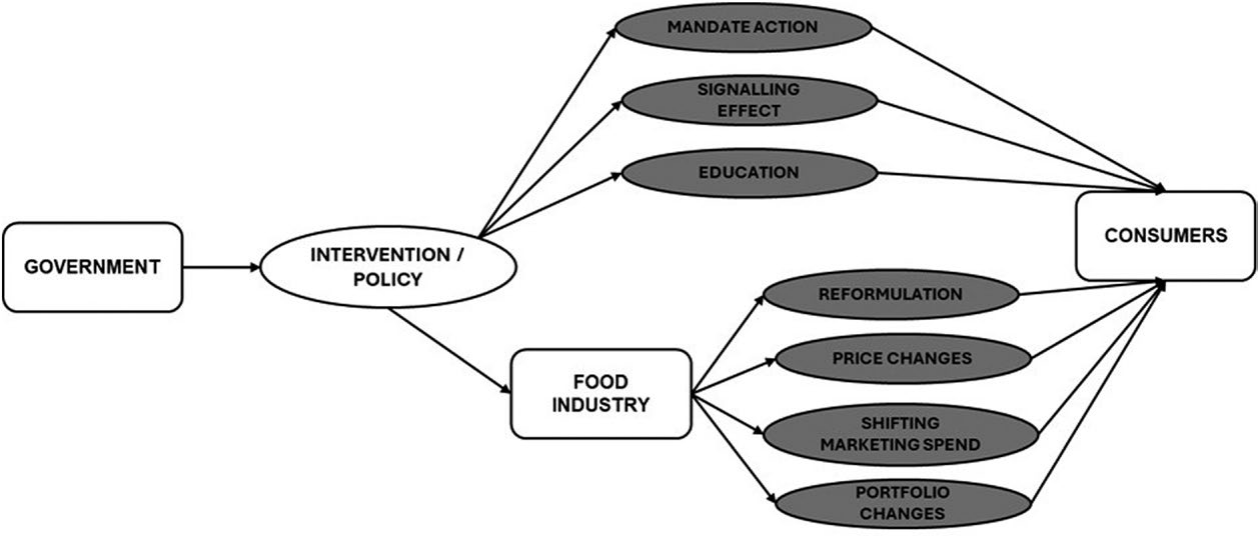
: The triad of actors in food policy implementation.

To properly recognize the effects of these interventions and thus conduct an adequate ethical analysis of the intervention, it is necessary to recognize the role of industry in implementation. To do this, we suggest that ethicists adopt a ‘triad’ approach which recognizes both the ethical relationships between government and consumers (which, as discussed in the introduction, are the primary concern of existing ethical analysis), as well as relationships between government and industry, and critically between industry and consumers (see Figure 2 which provides a simplified iteration of what such a framework might look like). We note that these relationships can be bidirectional, particularly where policy-driven changes in purchasing and consumption behaviours by consumers result in industry actors having to change their own behaviours and products. The bidirectionality and the full ambit of these complex relationships are outside the scope of this article and for this reason, the complexity that exists is not fully reflected in Figure 2 for simplicity’s sake. However, we would envisage that in conducting an ethical analysis of specific interventions, these complexities could be investigated and expanded upon.

Prior work has recognized the powerful role of the food industry in shaping policy, discourse and scientific evidence based on nutrition—and in doing so, its undermining of public health as an ethically salient consideration (Azevedo and Vartanian, 2015). Industry is often a powerful actor in public health nutrition policy and may exert substantial influence over the policy landscape (Nestle, 2002; Gómez, 2024). It is noted that this influence may not be neutral or positive towards public health as there is evidence showing how the industry may attempt to dilute and circumvent the public health aims of a policy rather than merely being a passive recipient and implementer of government’s food policies (Nestle, 2002; Vandenbrink *et al.*, 2020; Erzse *et al*., 2022). This further highlights the ethical relevance of the food industry’s conduct. Others have recognized the powerful role of the food industry in shaping policy, discourse and scientific evidence based on nutrition—and in doing so, its undermining of public health as an ethically salient consideration (Azevedo and Vartanian, 2015). These further buttress the importance of considering industry as an active role player in the ethical analysis of food policy and are issues worth considering within specific contexts and analyses. The ethics of the food industry’s conduct in relation to these policies and broader public health is clearly salient but we do not purport to make any determinations about the conduct more broadly. Rather, we suggest that by adopting the wider framing we propose, these issues could be properly considered to develop a comprehensive and contextually responsive ethical analysis of the food policies.

This triad of obligations and this recognition of industry as an intermediary in the implementation of these policies does not erase the relationship between governments and their citizens. That is to say, the existing approach of considering whether the government’s conduct is ethical should not be erased under this framing but rather, be supplemented. There remain certain impacts from these policies that emanate directly from the government’s decision to implement them or from the interventions themselves. For example, the adoption of a tax on sugary beverages and many other measures targeting unhealthy food may have a ‘signalling effect’ on consumers that the product being taxed is harmful and this can lead to reductions in consumption in itself (Alvarado *et al*., 2021). When an interpretive labelling scheme is implemented, consumers may experience certain negative emotions from seeing the label on food products that carry it, which are shaped by industry decisions. Yet, governments may also adopt public education campaigns alongside these restrictive interventions, which may result in negative outcomes for a consumer, including the stigmatization of obesity and social shaming for purchasing unhealthy foods. These direct outcomes of policy implementation ought to be considered in any comprehensive ethical analysis of the policies but they should not be the *only* outcomes considered nor should the responsibility of implementing these interventions be attributed solely to the government.

We suggest ethicists reframe their analysis of policies to recognize the variety of potential outcomes that may result from a particular policy and engage in a nuanced analysis of these differing outcomes.

## IMPLICATIONS OF INCLUDING FOOD PRODUCERS AND OTHER FOOD INDUSTRY ACTORS IN ETHICAL ANALYSIS OF UNHEALTHY FOOD POLICIES

This reframing of the approach to ethical analysis of food policy has several implications for how ethical analysis ought to be conducted.

First, there should be recognition of the complexity and diversity of outcomes a policy may have, which requires us to reconsider some of the assumptions upon which we build ethical evaluations. At the outset, the assumption that a policy restricts or disincentivizes particular consumer choices is no longer sound as it is not a certainty that these policy measures will translate into these outcomes. This will affect arguments for and against the policy on the basis of ethical considerations like choice, equity and effectiveness. This can be seen in the example of an SSB tax.

Rather than assuming that SSB tax results in a direct price increase on beverages, ethicists should recognize the variety of potential responses that industry actors may have. Instead of a price increase on the taxed products, an SSB tax may result in a producer introducing more untaxed products, making changes to product sizes, removing heavily taxed products or changing the composition of particular products (to add artificial sweeteners or reduce the sugar content) (Scarborough *et al*., 2020; Forde *et al*., 2022; Alvarado *et al*., 2023). Industry actors may also absorb the cost of the tax or increase prices of untaxed products so there is no price difference between taxed and untaxed products (Alvarado *et al*., 2023). These results have different impacts on consumers’ health, finances and choices, as well as their ability to enjoy their preferred beverages, and thus the ethical analysis for each is different. The evaluation of the UK Soft Drinks Industry Levy (SDIl) provides a good example of the development of a systems map that illustrates the multiple mechanisms through which an SSB tax may operate (National Institute for Health and Care Research, 2017). We note that while some responses were foreseen, others were unexpected and only identified during evaluations, with implications for the timing and ongoing nature of the corresponding ethical analysis.

Depending on the industry response, the direct public health aim of an SSB tax—price increases reducing consumption and thereby improving health—may not be fully realized. If industry absorbs the cost of the tax or increases the prices of untaxed products so there is no price difference between taxed and untaxed products, this would undermine the public health benefit of the tax. However, if manufacturers reduce the sugar content of SSBs, this means that the price of SSBs does not increase by the full amount of the tax and thus, the associated reductions in consumption of SSBs may not follow, but instead, there may be less consumption of sugar. Whether this has a better or worse effect on health than price increases depends on empirical facts about how much sugar is decreased, what, if anything, is used to replace sugar and any attendant health impacts and how much-increased price would have reduced consumption and for whom.

Similarly, depending on the industry response, an SSB tax may result in a reduction in the products available for purchase; the ethical analysis of the tax, then, should not assume that it merely disincentivizes certain choices but does not eliminate them. On the other hand, an SSB tax may result in more products being made available, thereby increasing consumer choice, which should also be registered in the ethical analysis of a tax.

The same point holds for equity-based ethical considerations. On the assumption that an SSB tax will increase the price of the taxed products, such a tax is sometimes objected to as financially regressive and therefore inequitable towards low-income people (though progressive from a public health standpoint). However, if the industry opts instead to remove heavily taxed products from the marketplace or to reformulate products to contain less sugar, without changing product prices, the tax does not have this regressive effect. This is an important factor in ethical analysis which may ultimately bear on whether a policy is ethically justifiable and permissible.

In addition, this diversity of outcomes and the uncertainty in how the industry will respond places an additional ethical burden on the government to manage, to some extent, the uncertainty or account for differing outcomes in its policy design. Thus, governments may need to engage in a more sophisticated analysis which recognizes a range of foreseeable potential outcomes before the policy is implemented. One mechanism by which governments create greater certainty in outcomes is performance-based regulations which seek to achieve a specified outcome (such as reduction in sodium content of foods) without specifying to industry how this outcome ought to be achieved. Alternatively, governments may need to revise the proposed measure to try to avoid ethically unacceptable outcomes. However, there are practical limitations to a government’s ability to do this. We acknowledge that there may be outcomes which are not foreseen by policymakers prior to implementation.

Second, in expectation of innovation (and uncertainty in the case of more novel interventions), ethical analysis needs to become a dynamic, ongoing exercise that is conducted over the course of policy development, implementation and evaluation. Rather than limiting consideration of ethical analysis to the pre-implementation stage of an intervention, there is a need to conduct ongoing ethical evaluations of a policy as we learn more about how the industry has responded to the measure and the effects these responses have on consumers. Importantly, policies should also be evaluated and potentially revised in light of the ongoing developments in how they are implemented and the effect they may have on health outcomes—following the kind of adjusting the compass-bearing approach as to whether to continue or amend policies (Ogilvie *et al*., 2020). If a particular mechanism for policy implementation via industry is ethically preferred or is to be avoided, the policy may require adjustment or revision to better promote that preferred mechanism or to ameliorate unintended negative consequences of the policy (Ogilvie *et al*., 2020). This is something that can only be achieved if there is an ongoing ethical analysis of policies and a consideration of the ethical implications of policy implementation when policies are being revised. In turn, it may be necessary to consider reframing the initial ethical analysis of a policy as a more modest, preliminary analysis of the policy rather than a definitive determination of the ethical acceptability of the policy.

Third, our analyses need to recognize members of the industry as autonomous agents who are making decisions that, in themselves, may also require ethical analysis. The degree to which industry responses to a policy may influence its impacts on health and consumers raises further questions about the ethical responsibilities the industry has towards consumers, and how the industry should be held accountable for fulfilling them. As ethicists, we should consider what standards industry actors should be evaluated against as part of a comprehensive ethical analysis of these policies. Those conducting analysis may be required to evaluate the range of potential policy responses industry actors may adopt and consider which of these are more ethically acceptable or be preferred over others. However, the recognition of this triad of actors and the significant role of industry in shaping the impact of these policies does not absolve the government from ethical responsibility for the effects of its policies, including both the direct effects of policies and the indirect effects of policies that are mediated by industry implementation. The influence governments have on individual choice remains highly relevant to any analysis of food policies.

## CONCLUSION

In conclusion, recognizing the complexity of restrictive food policies and the multifaceted interactions among governments, consumers and the food industry that result from these interventions is essential for conducting comprehensive ethical analyses. Not only does this more comprehensive approach enable consideration of a greater range of the responses to food policy interventions but it also enables ethical analyses to be more responsive to developments on how these novel interventions are operationalized by actors in the food industry. Moving forward, it is imperative for ethicists analysing these interventions to engage on an ongoing basis, and for policymakers to consider these ongoing analyses as part of their monitoring and evaluation of policies.

## Data Availability

No new data were created during the development of this perspective article.
